# Metformin: A Potential Candidate for Targeting Aging Mechanisms

**DOI:** 10.14336/AD.2020.0702

**Published:** 2021-04-01

**Authors:** Die Hu, Fangfang Xie, Yongwei Xiao, Chen Lu, Jianing Zhong, Defa Huang, Jie Chen, Jifu Wei, Yu Jiang, Tianyu Zhong

**Affiliations:** ^1^The First School of Clinical Medicine, Gannan Medical University, Ganzhou, China; ^2^Laboratory Medicine, First Affiliated Hospital of Gannan Medical University, Ganzhou, China; ^3^Key Laboratory of Prevention and Treatment of Cardiovascular and Cerebrovascular Diseases, Ministry of Education, Gannan Medical University, Ganzhou, China; ^4^Precision Medicine Center, First Affiliated Hospital of Gannan Medical University, Ganzhou, China; ^5^Department of Pharmacology and Chemical Biology, University of Pittsburgh, Pittsburgh, PA 15261, USA

**Keywords:** metformin, aging, Insulin-Like Growth Factor I, oxidative stress, proteostasis

## Abstract

Aging is a universal phenomenon in all biological organisms, defined by the loss of reproductive capacity and a progressive decline in fitness. In humans, aging is further associated with an increased incidence of disease conditions. The current aging population has become a primary public burden of the 21^st^ century. Therefore, to delay the aging process and maintain fitness in the aging population, the discovery of novel anti-aging drugs remains an urgent need. In recent years, metformin, a widely used hypoglycemic drug, has attracted growing attention in the field of anti-aging research. Reportedly, numerous studies have indicated that metformin regulates aging-related pathways, possibly delaying the aging process by modulating these pathways. The elucidation of these anti-aging effects may provide insights into the age-retarding potential of metformin. The present review focuses on the predominant molecular mechanisms associated with aging, as well as the anti-aging effects of metformin.

With advancements in health care and medical technologies, the human life span has been consistently growing. However, the downside of this health prosperity is an increasingly aging. According to the World Health Organization (WHO), between 2015 and 2050, the proportion of the population aged over 60 years will increase from 12% to 22% worldwide [1], translating into a population of 2 billion. In addition to reduced fitness, aging is a major risk factor for numerous age-related diseases, including diabetes [2], cancer [3], neurodegenerative diseases [4], and cardiovascular disorders [5].

Notably, the rate of human aging is influenced by genetic and environmental factors expressed through changes in various cellular signaling pathways and metabolic processes, including oxidative stress [6], telomere attrition [7], epigenetic alterations [8], proteostasis, autophagy and mitochondrial functions [9]. Furthermore, it remains controversial whether aging is a programmed process. However, recent studies investigating several metabolism altering drugs, including metformin [10], rapamycin [11], resveratrol [12], spermidine [13], curcumin [14] and astaxanthin [15], have suggested that while the aging process, cannot be rewired, it could be delayed. Importantly, these drugs have served as molecular tools, allowing us to examine pathways and mechanisms impacting the aging process. In this review, we will focus on metformin, a first-line drug used for the treatment of type 2 diabetes, and assess its anti-aging effects, as well as underlying mechanisms.

## Metformin

Metformin, a biguanide derivative, is the first-line drug indicated for the clinical treatment of type 2 diabetes, discovered in 1922, and introduced as a therapeutic agent in 1957 [16]. Metformin, initially extracted from the plant *Galega officinalis* (French lilac), contains two coupled molecules of guanidine with additionally substituted groups. Although metformin the hypoglycemic effect is inferior to other biguanides (phenformin and metformin), it is widely indicated owing to its safety (low lactic acidosis). In 2005, the International Diabetes Federation (IDF) metformin was used as a first-line hypoglycemic agent in type 2 diabetes [17].

Reportedly, the oral bioavailability of metformin is approximately 50%. In a patient with diabetes, following the oral administration of a 2.5 g does, metformin was first absorbed by the duodenum and ileum, and then exited intestinal cells through the organic cation transporter (OCT1) on the basolateral membrane, entering the portal vein and for absorption by the liver [18].In animals and humans, metformin remains unmetabolized and is eventually cleared by the kidneys. Positron emission tomography (PET) of 11-C labeled metformin revealed that the drug was mainly enriched in human and mouse livers [19]. Currently, it is widely accepted that metformin entering the liver cells mainly accumulates in the mitochondria and participates in mitochondrial activity, which may be associated with the positive charge of metformin.

## Mechanism underlying the anti-diabetic effect of metformin

As a reversible non-competitive inhibitor, metformin interacts with the ND3 subunit of the mitochondrial respiratory chain complex, undergoing conformational changes, and inhibiting the electron transport chain (ETC), thus impacting ATP synthesis [20]. In cells, decreased ATP generation causes increased ADP/ATP and AMP/ATP ratios, ultimately activating the AMP-activated protein kinase (AMPK).

For metformin to exert its anti-glycemic effect, AMPK is a key molecule. Activated AMPK phosphorylates the transmitter of regulated CREB activity 2 (TORC2), preventing its entry into the nucleus, and decreasing the expression of peroxisome proliferator-activated receptor-gamma coactivator 1alpha (PGC-1α). PGC-1α is a transcriptional coactivator of CREB (cAMP response element-binding protein), and acts downstream of the nuclear receptor, hepatocyte nuclear factor 4α (HNF4α), and the transcription factor Foxo1, promoting the expression of gluconeogenic enzymes [21]. Subsequently, AMPK activation by metformin increases SHP gene expression, which in conjunction with PGC-1α, plays an important role in the inhibiting heterogeneous glycogen gene expression [22]. Additionally, activated AMPK increases the biological’s sensitivity to insulin. Reportedly, long-term metformin treatment (clinical dose) accelerated the oxidation of hepatic fat by suppressing the expression of SREBP-1 (a key lipogenic transcription factor), acetyl-CoA carboxylase 1 (Acc1), and Acc2, boosting insulin sensitivity [23, 24]. Conversely, inhibition of the mechanistic target of rapamycin complex 1 (mTORC1) pathway (the TSC1/2-mTOR signaling pathway) may be a contributing factor in enhancing insulin sensitivity. Furthermore, AMPK phosphorylates TSC2 and increases its inhibitory activity toward Rheb, a proximal activator of mTORC1. This reduces insulin receptor substrate (IRS) serine phosphorylation, increasing IRS-dependent insulin signal transduction, consequently enhancing Insulin sensitivity [25]. Additionally, metformin reduces the transport of the RagA-RagC GTPase heterodimer through the nuclear pore complex (NPC) by inhibiting the respiratory capacity of the mitochondrial complex,, which further blocks mTORC1 activation [26]. Interestingly, inhibition of the TSC-mTOR signaling pathway restored cardiac autophagy in the diabetic heart by phosphorylating and activating the Unc-51 like autophagy activating kinase (ULK1) complex, decreasing the cardiac accumulation of polyubiquitinated proteins and abnormal mitochondrial aggregates, thereby protecting the cardiac structure and function [27].

## Effects of metformin on aging

In addition to the established therapeutic indications in the treatment of diabetes, metformin has demonstrated have beneficial effects in several aging related diseases. In a study investigating the effect of metformin on complications in overweight patients with type 2 diabetes, metformin reduced the incidence of cardiovascular events and all-cause mortality in patients with diabetes when compared with insulin-treated patients [28], extending the survival of diabetic patients by 38% [29]. Additionally, metformin has reported potential as an agent in Parkinson's disease [30]. In animal studies, metformin extended the lifespan of mice [31-35], as well as the survival time in age-related diseases [36]. Similarly, investigations undertaken in the C. elegans aging model [37, 38]. All of above research have been listed in Table 1. Revealed the anti-aging effects of metformin, resulting in the approval granted by the Food and Drug Administration for use in clinical anti-aging trials. However, the mechanism by which metformin delays aging has remains elusive. The present review summarizes the main anti-aging mechanisms and proposes the potential targets of metformin in these anti-aging mechanisms.

**Table 1:** Metformin improves the health and longevity of different species.

Species	Model	Result
Worm	*C. elegans*	Metformin (25, 50, and 100 mM) increased mean lifespan (by 18%, 36%, and 3%) [37].
	*C. elegans*	Metformin at 50 mM increased median survival by 40% [38].
Mice	Female SHR Mice	Increased mean lifespan by 14% and maximum lifespan by 1 month [31].
	Female SHR Mice	Increased mean lifespan by 37.8% and maximum lifespan by 2.8 months (+10.3%) [33].
	HER2/neu mice	Increased mean lifespan by 8% and mammary adenocarcinoma latency by 13.2% [32].
	129/Sv Mice	Decreased the mean lifespan of male and female mice by 13.4% and 4.4% [34].
	Adult male C57BL/6 mice	Increased extension of mean lifespan by 5.83% [35].
	Male Huntington's disease mice	Prolonged the survival time, increased lifespan by 20.1% [36].
Human	Diabetic patients	Risk reduction of 32% for endpoint, 42% for mortality [28].
	Diabetic patients/non-diabetic patients	Metformin monotherapy increased median survival time by 15% and 38% compared with normal and sulphonylurea monotherapy, respectively [29].
	Parkinson's disease patients	Reverses mitochondrial unction [30].

## Anti-aging mechanisms

**Figure 1.**
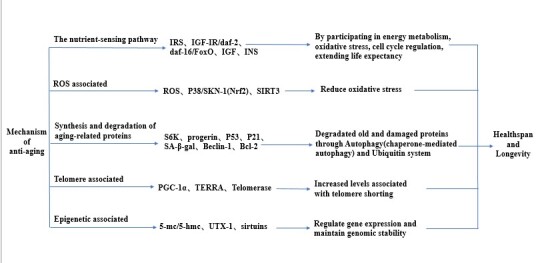
Possible mechanisms associated with lifespan. Summary of various possible pathways directly or indirectly involved with health and longevity. Certain targets play important roles in the anti-aging effects of metformin. The mechanism of action of these targets is specifically introduced through relevant experiments, which provide a basis for the anti-aging effects of metformin.

Aging is a malleable process that is regulated by a variety of signaling mechanisms, including nutritional sensing pathways, reactive oxygen species (ROS)-regulated oxidative stress, protein homeostasis, telomeres, and epigenetics (Fig. 1). Changes in the signaling activities of the pathways, either by genetic mutations, epigenetic modifications or environmental stimulations, can accelerate or slow the aging process. These mechanisms are thus referred to as anti-aging mechanisms for their association with aging.

*The nutrient-sensing pathway mediated by insulin/insulin-like growth factor-1 (INS/IGF-1) signaling (IIS)*. Nutrient-sensing is a major mechanism regulating nutrient uptake and signaling, affecting multiple signaling and metabolic processes. The IIS pathway, consisting of the INS/IGF-1 receptor, is a key in this process. In the mouse brain, persistently low IGF-I levels contributed to prolonged lifespans and reduced age-related mortality in animals [39]. Furthermore, downregulation of IRS2, an adaptor protein of the IGF-1 receptor in the whole body or brain, could extend the increased mouse lifespan by up to 18% [40]. This extended lifespan phenomenon was associated with the reduced secretion of IGF-1 owing to an increased association between brain IGF-1 receptors and IGF-1. Additionally, the impairment of IR/IRS2 signaling reduced β-amyloid accumulating in the brain of mice presenting Alzheimer’s disease, thus demonstrating improvement in this aging-related disease [41]. Elevated insulin contributes to age-dependent insulin resistance, ultimately resulting in aging. Therefore, improving insulin sensitivity by decreasing insulin levels can extend lifespan [42].

In *C. elegans* DAF-2 and its downstream effector, DAF-16, are critical regulators of the IIS pathway, which are homologs of the IIS tyrosine kinase receptor and forkhead/winged-helix-box O (FOXO) transcription factors, respectively [43-45]. A previous study revealed that mutations in DAF2 extended the lifespan of affected worms [46], which could be reversed by DAF-16 knockdown. DAF-16 is a negative regulator of the insulin-like gene *ins-7*, a putative DAF-2 agonist [43]. *Downregulation of DAF-16 increases ins-7 expression and consequently, DAF-2 signaling. Thus, it has been suggested that* reduced insulin levels increased the worm lifepan [47]. Consistent with this hypothesis, activated IIS increased the phosphorylation of DAF-16, preventing DAF-16 from entering into the nucleus to regulate its target genes [48, 49], ultimately impacting worm lifespan. Collectively, the discussed studies establish the IIS pathway as a key signaling event in controlling longevity in both mammals and worms.

*ROS associated aging*. ROS are byproduct of various cell compartments, including the endoplasmic reticulum (ER), mitochondria, and peroxisomes, primarily produced during operation of the mitochondrial ETC. Harman first proposed the free radical theory of aging based on the hypothesis that mitochondrial dysfunction leads to oxidative stress, further promoting age-related diseases via induction of ROS production [50]. These diseases include diabetes [51], cataracts [52], atherosclerosis [53] and Alzheimer’s disease [54]. Generally, excessive ROS production readily induces oxidative modifications, damaging certain active molecules, including lipids, DNA, mitochondrial DNA (mtDNA), and proteins. For instance, ROS induces the peroxidation of lipids, which accumulate in the skin to form age spots. In mice, the depletion of mtDNA results in skin wrinkles and visual alopecia [55]. However, low concentrations of ROS can activate the SKN-1 (Nrf2 in mammals) pathway, a predominant pathway for cells to resist oxidative stress. Following induction by ROS, SKN-1 phosphorylation promotes nuclear translocation and induce transcribed genes during phase II detoxification. Hence, the expression of various antioxidant proteins is upregulated to reduce the level of oxidative stress, including glutamylcysteine synthetase (GCS), glutathione-S-transferase (GST), and superoxide dismutase (SOD). ROS production by the ER and mitochondria can cause rapid oxidation of cysteine residues within the transmembrane protein IRE-1 kinase active site through SKN-1, thereby inhibiting kinase activity and unfolding protein reactions [56], eventually promoting the lifespan of *C. elegans*.

Additionally, there exist unique defense mechanisms in organisms to maintain ROS homeostasis in order to delay aging. Mitochondrial sirtuin 3 (SIRT3) revealed significant effects on aging. SIRT3 is a class of NAD^+^-dependent deacetylase, maintaining the mitochondrial mass and bioenergetics by regulating the acetylation of mitochondrial proteins. Reportedly, the VNTR allele in intron 5 of human SIRT3 is closely associated with male longevity [57]. SIRT3 directly bind to serine hydroxymethyltransferase 2 (SHMT2, a key metabolic enzyme in one-carbon metabolism) and maintain its activity in the presence of glucose starvation, thereby contributing to mitochondrial ROS stress resistance and ensuring a supply of intracellular biomacromolecules [58]. Additionally, SIRT3 can enhance mtDNA4977 replication and transcription in chondrocytes [59], enhancing the mitochondrial antioxidant activity in endothelial cells by mediating the deacetylation of FOXO3-dependent mitochondrial antioxidant enzymes such as manganese SOD (MnSOD) and peroxiredoxin 3 (Prx3) [60]. Moreover, SIRT3 is a positive regulator of autophagy and can activate mitochondrial autophagy in tumor cells by promoting the interaction of the voltage-dependent anion channel 1 (VDAC1) with Parkin, a target of mitophagy [61]. Interestingly, ROS are necessary to activate autophagy. When oxygen homeostasis cannot be corrected by the antioxidant cellular machinery, autophagy is activated to remove damaged components [62]. Collectively, the above observations indicate that the biological’s antioxidant mechanism helps maintain a stable ROS state and delays aging. However, excessive ROS accumulation can activate autophagy to remove damaged molecules through mitoautophagy.

*Synthesis and degradation of aging-related proteins*. Aging individuals are characterized by an accumulation of aging-associated abnormal proteins, including progerin and senescence-associated β-galactosidase (SA-β-gal). Progerin is a mutant form of lamin A (lamin), associated with the occurrence of the Hutchinson-Gilford premature aging syndrome [63, 64]. SA-β-gal is a hydrolase enzyme activated in senescent cells and has been widely used as a reliable marker of these cells [65-67]. Additionally, oncoprotein p53 was reported as a vital regulator in cell cycle progression [68]. Permanent cessation of proliferation is a hallmark of senescence. During the early stages of senescence, the level and activity of p53 increases at the G2 phase, which transcriptionally upregulates p21, ultimately leading to cell-cycle arrest by impeding the formation of the CDK4-CDK6/cyclin D complex. At a relatively late phase of senescence, F-box only protein 22 (Fbxo22) is highly expressed in senescent cells, binding to p53 and lysine-specific demethylase 4A (KDM4A, a kind of demethylase) through its FIST-N and FIST-C domains. Fbxo22 acts in concert with KDM4A (SCF-Fbxo22-KDM4A complex) to couple the demethylation and ubiquitination of p53 for selective degradation. Downregulation of p53 is crucial for the induction of senescence-associated secretory phenotype (SASP) [69].

For aging-related proteins, homeostasis (including synthesis and degradation) is closely related to the biological anti-aging process. mTOR, a rapamycin target protein, plays a crucial role in the maintenance of protein homeostasis by modulating protein synthesis [70]. Inhibition of mTOR prolonged lifespan by downregulating downstream targets, such as ribosomal protein S6 kinase (S6K) or protein synthesis in yeast, worms, fruit flies, and mice [71, 72]. The downregulation of mTOR activity decrease the phosphorylation levels of ribosomal protein S6 kinase, translational elongation factor 2 (eEF2) kinase and translation initiation factor 4 binding proteins (4E-BPs), hence, reducing ribosome biogenesis and protein translation [73-75].

The efficient degradation of aging-related proteins is another strategy to maintain protein homeostasis. Particularly, autophagy is an essential approach to degrade abnormal proteins. Initially, the inhibition of mTOR activity induces the dephosphorylation of ULK1 (Atg1), activating ULK1 kinase to induces autophagy (formation of phagophore). In turn, ULK1 promotes the localization of autophagy proteins to phagophore through Beclin-1 (Atg6) phosphorylation, generation an LC3-phosphatidylethanolamine complex (LC3-PE complex) under the action of Atg5 and forming autophagosomes. Autophagosomes and lysosomes fuse to form autophagolysosomes, which ultimately degrade the engulfed contents [76]. In general, suppression of autophagy could causes the excessive accumulation of damaged proteins and induces cell death, accelerating aging and shortening lifespan [77-79]. Conversely, enhancing autophagy by overexpressing autophagy-associated gene 5 (ATG5) can significantly extend lifespan [80]. Notably, a correlation between longevity and Beclin1 expression, an essential mediator of autophagy activation, has been reported. Compared with healthy young people, elevated Beclin1 expression levels were detected in healthy centenarians, proposing that the prolonged lifespans were associated with activated autophagy [81]. Additionally, the ubiquitin-mediated protein degradation system (UPS) is another pathway to eliminate aging-related proteins or damaged proteins in cells. Various misfolded or unfolded proteins are usually transferred to the proteasome to undergo degradation after ubiquitination, which helps maintain protein homeostasis. Growing evidence indicated that enhancing UPS activity can delay aging through the removal of misfolded or damaged proteins. Furthermore, activation of ubiquitination by increased proteasome activity could improve the function of aged fibroblasts [4, 82], while downregulation of the E3 ubiquitin ligase of DAF-16, to disrupt ubiquitination, could revert extended aging in *C. elegans* [83]. In summary, restricting the production of aging-related proteins through mTOR inhibition, as well as enhancing the clearance of damaged proteins (autophagy, ubiquitination), can delay the aging process. In contrast, abnormal autophagy can result in the aggregation of aging-related proteins and damaged proteins, thereby inducing premature aging.

*Telomere associated aging processes*. A telomere is a repeated TTAGG sequence that is present at the end of linear chromosomes in eukaryotic cells. This sequence is involved in maintaining chromosomal stability by preventing the degradation of chromosomal ends or their fusion with adjacent chromosomes. Telomeres gradually shorten owing to cell division, and shorter telomeres may cause cellular senescence or apoptosis. Generally, the shortening rate of telomere is affected by oxidative stress and antioxidant defense [84, 85]. Previously, studies have reported that telomeric DNA is more reactive to ROS than non-telomeric sequences. Antioxidants can decrease ROS production, thus reducing telomere shortening and protecting against chromosomal aberrations of telomere origin [86, 87]. Moreover, telomerases and certain cytokines can maintain telomere stability in cells. Telomerase is a reverse transcriptase that maintain telomere length by filling in telomeres lost during DNA replication.

*Epigenetics and aging*. DNA methylation and histone modifications are the two main types of epigenetics. Recent evidence has proposed a link between epigenetics and cellular lifespan. DNA methylation refers to the binding of a methyl group to cytosine (5-methylcytosine, 5-mC) at the 5’ end of CpG islands through DNA methyltransferases. Reportedly, the hippocampus of patients with Alzheimer’s disease demonstrated significantly decreased levels of 5-mC and 5-hydroxymethylcytosine (5-hmC) [88]. during human aging, DNA methylation is tissue-specific. Therefore, the aging of cells can be predicted through the level of DNA methylation at a particular CpG site [89]. The Ten-eleven translocation (TET) protein is an important enzyme in DNA demethylation, catalyzing the conversion of 5-mC to 5-hmC. Additionally, the anticancer effects of TET have attracted considerable interest and may be associated with aging and aging-related characterization. Gontier *et al*. [75] have observed that TET2 and 5-hmc were significantly reduced in the hippocampus of older animals. In the mature adult hippocampus, TET2 amelioration rescued age-related neurodegenerative decline. This phenomenon could be attributed to TET-mediated to and inhibitor of growth family member 1 (ING1), which induce site-specific demethylation to regulate C/EBP-dependent sexual processes regulating metabolism and aging [76].

**Figure 2.**
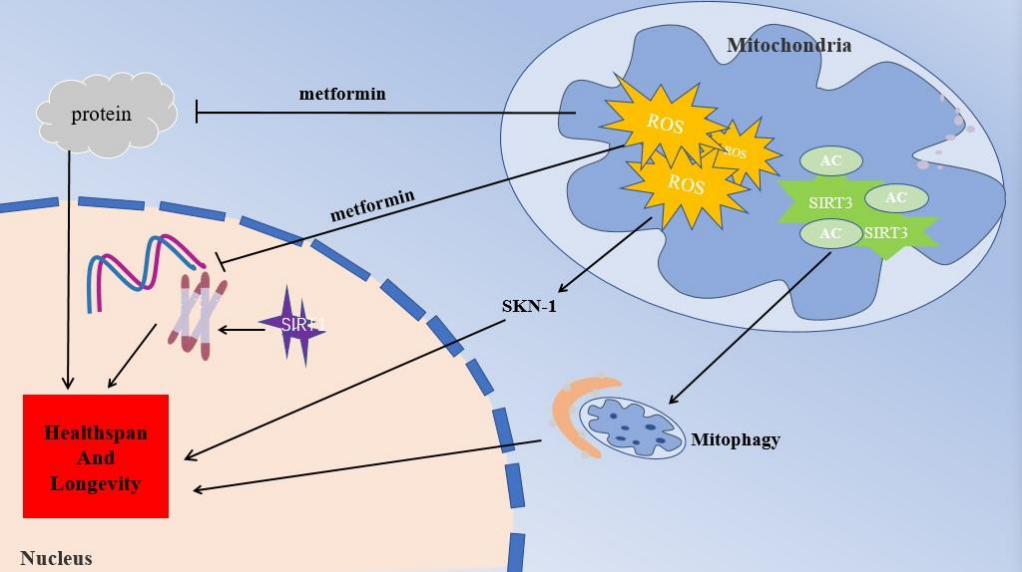
Metformin delays aging by reducing oxidative stress. Metformin protects biological macromolecules such as proteins and DNA by reducing excessive ROS production in the mitochondria. Furthermore, metformin can activate the SKN-1 pathway through ROS, which acts as a signaling molecule to delay aging. In addition, metformin activates SIRT3, participates in mitochondrial autophagy and reduces oxidative stress, thereby delaying cell senescence. ROS, reactive oxygen species; SIRT1, sirtuin 1; SIRT3, sirtuin 3.

Histone modification refers to the modification of histones by methylation and acetylation under the action of associated enzymes. Histone modification affects the binding of histones to DNA, which in turn alters gene expression. In nematodes, H3K27 demethylase activity was enhanced by senescence, affecting IIS signaling and reducing H3K27me3 expression [90]. Another studyreported that the H3K27 methylase konckdown extended the lifespan in *Drosophila* [91]. Sirtuins, a family of deacetylases proteins essential in regulating histone acetylation, play a crucial role in the aging process. Reportedly, sirtuins control stress tolerance and longevity in nematodes and *Drosophila* [92, 93]. Additionally, mice administered sirtuin activators, SRT2104 and SRT1720, demonstrated a longer lifespan than control mice [94]. Various acetyltransferases, including SIRT1, SIRT6, and SIRT7, regulate genes. Sirt1 contributes to the deacetylation of key histone lysine residues, including H3K9 and histone H4K16. Moreover, Sirt1 can reduce p53-mediated transcriptional activity and maintain genome integrity [95]. Epigenetic modifications are expected to be potential therapeutic strategies for anti-aging and aging-related diseases (Fig. 2).

## Mechanisms underlying the anti-ageing effect of metformin

*Metformin delays aging through the IIS pathway*. As discussed earlier, hyperglycemia and hyperinsulinemia accelerate aging. Both conditions are regulated by the IIS pathway. Notably, hepatic insulin sensitivity depends on the fenestration porosity of liver sinusoidal endothelial cells (LSECs) [96]. In mice treated with metformin for 9 months, enhanced fenestration porosity and frequency were observed, increasing insulin sensitivity in the liver and prolonging lifespan. This is related to the activation of pAMPK, eNOS / cGMP and pMLCK / actin remodeling pathways by metformin [97]. Furthermore, in middle age mice, long-term metformin treatment improved physical performance and increase insulin sensitivity, thus extending lifespan [35]. Additionally, metformin enhanced cognitive function in animal models of cognitive impairment by reducing IRS2 and IGF1R in neurons, thus preventing the aging phenotype [98].

The accumulation of advanced glycation end products (AGEs) is considered a critical marker of aging [99], formed by the combination of accumulated glucose with other proteins. Increased glucose levels cause normal proteins to lose their biological functions by transforming them into aging proteins. Metformin reduces the accumulation of AGEs by facilitating glucose utilization in the tissue. This could lower AGEs in patients with type 2 diabetes (T2D) and polycystic ovary syndrome (PCOS), and increase receptors of AGEs [100]. This phenomenon could enhance the interaction between AGEs and their receptor (RAGE). Upon binding, intracellular oxidative stress and nuclear factor-κB signaling pathways are activated to regulate transcription levels of endothelin-1 and tumor necrosis factor (TNF)-α, thus, delaying cell senescence.

*Metformin delays aging by regulating ROS levels*. Reportedly, metformin reduces the production of mitochondrial ROS through the reverse electron flow of the mitochondrial respiratory chain complex Ⅰ, which plays a critical role in extending life. In lens epithelial cells (LECs), metformin reduced the mitochondrial potential and ROS levels, as well as inhibited cell senescence [101], similar effects were observed in the brains of naturally aging rats and D-galactose-induced aging rats [102]. Furthermore, chronic low-dose metformin upregulated ER-localized glutathione peroxidase 7, thereby protecting worms and humans from premature aging [103].

In addition to reducing intracellular ROS, metformin can produce an opposite effect on ROS production. De Haes [104] observed that metformin elevated ROS levels, which activated the transcription of SKN-1, a longevity-promoting factor in worms. In *C. elegans* with mutant SKN-1, this metformin effect disappeared [38]. As mentioned earlier, SKN-1 is an important anti-aging molecule, that can be activated by ROS. This finding may be related to the inhibition of metformin by the IIS pathway. Evidence indicates that IIS inhibition resulted in increased ROS production, promoting SKN-1 activity [105].

Metformin can relieve the intensity of oxidative stress by upregulating SIRT3 levels in the mitochondria. In metformin-treated HAEC cells, AMPK activation enhanced H3K79 methylation and induced an increase in SIRT3 levels, thereby delaying endothelial senescence [106]. Metformin inhibited oxidative stress and interleukin (IL)-1β-induced osteoarthritis-like inflammatory changes by enhancing the SIRT3/PINK1/Parkin signaling pathway [107]. In short, physiological concentrations of ROS can activate SKN-1 to delay aging, and excessive ROS can cause oxidative stress damage to cells. Metformin stimulate the production of physiological ROS concentrations to activate SKN-1; However, when ROS is excessive, it can delay cell senescence by reducing the level of oxidative stress (including inhibiting mitochondrial respiration to produce ROS and increasing SIRT3 levels).

*Regulation of protein homeostasis*. Notably, the regulation of protein homeostasis is the main mechanism of metformin-improved lifespan. A previous study reported that the expression of global progerin cells was reduced after metformin treatment in human mammary carcinoma [108]. The progerin protein is a mutation of nuclear-envelope protein lamin A, and induces early cellular senescence, associated with increased DNA-damage signaling [109]. Metformin decreased the translation of the progerin protein by inhibiting mTORC1 negatively regulating the phosphorylated S6 kinase1 (p70S6 kinase 1), ribosomal protein S6 and eIF4E binding protein 1(4E-BP1), thereby delaying senescence. Additionally, metformin promoted the clearance of progerin induced by the activation of AMPK/mTOR/ULK1-induced autophagy, thus prolonging the lifespan of fibroblasts [110]. Moreover, metformin can delay aging by inducing the production of certain aging-related proteins. For example, elevated glutathione peroxidase 7 (GPx7) expression was detected in metformin-induced human diploid fibroblasts (HDFs). GPx7 was localized in the ER and could prevent damage induced by oxidative stress, thus enhancing longevity [111]. Reportedly, age differences can influence the anti-aging effects of metformin. This difference was associated withlower levels of phosphorylated S6K1 (p70S6 kinase 1), rpS6, and 4E binding protein 1 (4E-BP1) in younger individuals, with no significant reduction observed older individuals [112]. Recently, Kulkarni *et al* [113] performed a randomized double-blind trial with metformin on patients >70 years old and revealed that metformin impacts tissue-specific transcriptomic changes and improves age-related metabolic disorders. The effects may be attributed to metformin regulation of aging-related upstream transcriptional regulators, including mTORC1, MYC, and TNF.

Another metformin mechanism attributed to improved aging is the activation of autophagy via inhibition of the mTOR signaling pathway. In Drosophila, metformin delayed senescence in intestinal stem cells (ISC) [114]. However, the anti-senescence effect of the metformin diminished upon Atg6 downregulation, suggesting that Atg6 (Autophagy-related gene 6, Beclin1 in mammals) plays a pivotal role in the effects of metformin. In yeast, Atg6 combines with Vps34, Vps15, and Atg14 to form Ptdlns 3-kinase complexes, which function in autophagy. Human Beclin 1 shares a 24.4% identity with ATG6/Vps30, forming a Bcl-2-Beclin 1-PtdIns 3-kinase-UVRAG multiprotein complex to act on the autophagy function [115]. In the D-galactose-induced rat brain, as well as naturally aged rat brain, metformin attenuated senile nerves by upregulating Beclin-1 expression, thereby improving senescence of neural cells [102]. A similar finding was reported by Feng and coworkers, revealing that metformin triggered autophagy was attributed to decreased Bcl-2 expression, increasing Beclin-1 through STAT3 (signal transducer and activator of transcription 3) inactivation [116]. In some cases, metformin treatment decreased the phosphorylation status of RSKS-1 (the human ribosomal protein S6 kinase B1 (S6K) homolog in C. elegans.), while increasing the nuclear localization of HLH-30 (the mammalian transcription factor EB (TFEB) orthologue).These findings indicate that downregulated mTOR enhances lysosomal biogenesis to regulate autophagy and lysosomal pathway-related gene expression [117, 118], resulting in delayed aging. Moreover, metformin augments the autophagy flux of D-galactose-induced LECs to delay the senescence of LECs [101].

**Figure 3.**
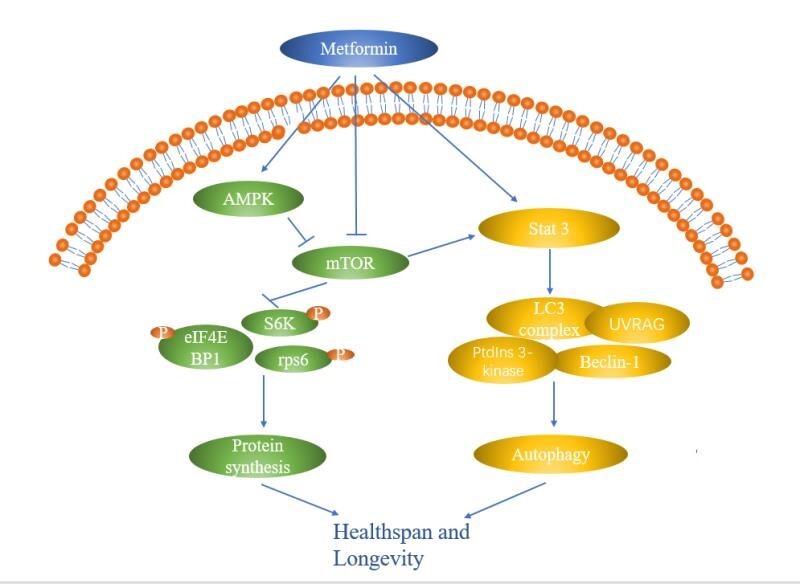
Metformin targets proteins involved in homeostasis and aging. Metformin downregulates mTOR to maintain protein homeostasis, including regulation of protein synthesis and degradation of damaged proteins via autophagy, which are major biological processes associated with aging.

In summary, metformin modulates ageing-related protein synthesis by regulating AMPK/mTOR signaling, enhancing autophagy to increase aging-related protein’s degradation, therefore, alleviating cellular senescence (Fig. 3).

*Metformin delays aging through telomeres*. Recently, metformin demonstrated superior on maintaining telomere stability. Garcia-Martin *et al* [119] indicated that metformin prevented placental telomere attrition. In the metformin-treated group, patients with diabetes mellitus demonstrated significantly longer telomeres than those in the intervention group. Similarly, in patients with diabetes treated with metformin, the telomere length of mononuclear cells was significantly increased compared with that in the placebo group [120]. Additionally, metformin activated endonuclease RAG1 via AMPK, which acts as an endonuclease, promoting DNA division and translocation, and ultimately activating telomerase [121]. Although the current mechanism by which metformin maintains telomere stability remains unclear, evidence supports an association between inhibition of oxidative stress and regulation of telomere transcription. Moreover, metformin can activate telomere transcription via SIRT1. SIRT1 is a positive regulator of telomere length in SIRT1-derived/SIRT1-deficient mice, and SIRT1 overexpression increases homologous recombination through telomeres, centromeres and chromosome arms [122]. SIRT1-mediated deacetylation induces the activity of PGC-1α, regulating telomere transcription in combination with Nrf1 [123]. Collectively, metformin could delay the aging process by maintaining telomere length.

*Metformin delays aging through epigenetic mechanisms*. Abnormal methylation frequently leads to genomic instability and diseases and reversing the abnormal DNA methylation status via TET activation could be a viable ‘rejuvenation’ strategy. According to Wu *et al*. [124] metformin can enhance the stability of TET2 and 5-hmc by activating AMPK-mediated TET2 phosphorylation. Reportedly, TET enzyme-mediated DNA demethylation was associated with the promotion of gene repair and maintaining genome integrity. Mechanistically, 5-hmc is locally deposited by the TET2 enzyme at the DNA damage site of HeLa cells, and the TET2 enzyme effectively repairs Aph-induced DNA damage during division [125]. Accumulation of DNA damage is one of the hallmarks of cellular senescence.

Additionally, metformin can affect the aging process via deacetylases. Previously, studies have reported that metformin can activate SIRT1 to improve renal outcomes, owing to the SIRT1 involvement in the pathogenesis of age-dependent and metabolic diseases [126]. Similarly, in cardiomyocytes, SIRT2 promotes AMPK activation by deacetylating kinase LKB1. Following SIRT2 knockdown, the cardioprotective effect of metformin was diminished [127].

Overall, metformin reduces oxidative stress to minimize DNA damage, promoting DNA damage repair by affecting the activity of epigenetic enzymes (TET2 enzyme, SIRT), eventually, enhancing genome stability. *Metformin delay aging through microbes*. As the gut is an important organ for metformin absorption, gut microbiota could be a major target mediating the anti-aging effect of metformin. Ahmadi et al. [128] observed that metformin promotes the formation of mucin and goblet cells via inhibition of Wnt signaling, reducing microbiota dysbiosis, reducing inflammation caused by intestinal leaks, and improving cognitive impairment in elderly mice. Moreover, the beneficial metabolites of gut microbes are of great interest in anti-aging, including agmatine. In apolipoprotein E-knockout mice (apoE-/-), exogenous agmatine can reduce atherosclerotic lesions by 40% [129]. In nematodes treated with metformin *E. coli* can enhance the production of agmatine through the PTS-CrP pathway, thereby promoting the host metabolism and prolonging lifespan [130]. Notably, CrP is a transcription factor targeted by metformin. However, the specific mechanism of agmatine is remains unclear owning to the lack of accurate methods to measure agmatime produced by microorganisms. Interestingly, Pryor et al [130] reported that glucose as an inhibitor of cAMP-CRP could affect the anti-aging effects of metformin through PTS-CrP. This provides possible evidence rationalizing why metformin does not affect *Drosophila* [131].

## Future directions of metformin in anti-aging

Notably, the regulatory effects of metformin on aging are convincing; however, the limitations need to be addressed. Supra-pharmacological concentrations were used in several investigations, even 10-100 times higher than the therapeutic concentration used in patients with type 2 diabetes [132]. It is challenging to accurately simulate human pharmacokinetics from quantitative administration in animal models. However, direct clinical trials face uncertain side effects owing to the high concentrations of metformin. Determining safe drug dosages and regimens is a primary goal for developing metformin as an anti-aging agent. Additionally, co-administration of metformin with other therapeutics is a popular theme for investigating the anti-aging effects. In a small trial, a cocktail of growth hormone, metformin, and dehydroepiandrosterone was investigated in nine healthy volunteers for one year. In these participants, the biological age was reduced by an average of 2.5 years, as measured by the GrimAge clock, which theoretically predicts human life expectancy [133]. However, clinical data regarding the age-retarding effects of metformin from large-scale and multi-center human clinical trials, are lacking. As of June 2020, among the 2,416 metformin applications registered at http://ClinicalTrials.gov, only 14 clinical trials concerned aging. Presently, research on the anti-aging effects of metformin mainly focuses on cellular experiments and some animal experiments, including nematodes and worms. The primary goal of the present research remains on verifying and confirming of desirable results reported in non-human studies.

## Conclusion

Delaying the process of aging has been an enduring goal of mankind. Current anti-aging research focus on nutrition sensing pathways, ROS, protein homeostasis, telomeres, epigenetics, and microorganisms. These pathways ultimately affect healthspan and longevity by affecting related gene expression, protein production, and enzyme activity. Additionally, these mechanisms are interconnected and often malleable, and the anti-aging effect of metformin is further related to these pathways.

Metformin, as an anti-aging drug candidate, has unparalleled advantages. It is inexpensive and relatively safe, with no obvious side effects observed during the 60 years of clinical application. Additionally, metformin provides superior cardiovascular protection, as well as anticancer and anti-inflammatory effects, essential indicators to measure healthspan. Regarding the anti-aging mechanism of metformin, the nutrient pathway is a key mechanism by which metformin lowers blood sugar levels and additionally mediates its anti-aging effects. Metformin reduces the level of AGEs, a marker of aging, by lowering insulin and blood glucose levels and increasing insulin sensitivity. ROS can damage the biostructure of macromolecules such as genes and proteins, accelerating aging. Nobably, metformin can stimulate the production of physiological ROS concentrations and activate SKN-1to delay aging. In the case of ROS accumulation, metformin can induce the production of antioxidant proteins (SIRT3, GPx7) to maintain ROS homeostasis and reduce the level of oxidative stress. Interestingly, the activation of autophagy and autophagic flux are regulated by ROS. Further, mTOR-mediated protein homeostasis plays a key role in delaying aging. Metformin inhibits mTOR activity by activating AMPK. Downregulation of mTOR leads to the activation of autophagy, preventing the accumulation of damaged proteins (for example, progerin and SA-β-gal). Furthermore, epigenetics and microorganisms are additional targets of metformin mediating anti-aging effects. Elucidating these mechanisms is crucial for understanding the biological effects of metformin and formulating efficacious strategies to delay human aging. However, exploring the anti-aging potential of metformin faces numerous challenges, including drug concentration, drug regimen, and lack of clinical trial data, thus necessitating future investigations.

In conclusion, a large number of studies have emphasized the effectiveness of metformin in age and age-related diseases, utilizing cell lines and animal models as a basis for their findings. Metformin, as a competitive candidate against aging, has demonstrated potential in preventing and treating senile diseases and improving health.
